# “Metrology is a key component of the industrial value-added chain”: an interview with Prof. Wolfgang Osten

**DOI:** 10.1038/s41377-022-00965-8

**Published:** 2022-09-22

**Authors:** Shuai Ding

**Affiliations:** Light Publishing Group, Changchun Institute of Optics, Fine Mechanics, and Physics, Chinese Academy of Sciences, 3888 Dong Nan Hu Road, 130033 Changchun, China

**Keywords:** Optical physics, Applied optics

## Abstract

Mendeleev said: “Science begins where one begins to measure,” and “exact science is unthinkable without measure.” During his more than 4-decades-long career, Prof. Wolfgang Osten has been devoted to optical metrology and inspection technologies. Osten and his research team developed the first automated system for the quantitative and qualitative evaluation of interferograms in the ‘80s, as well as the principle of tilted-wave interferometry for high-precision aspherical and free-form surface measurement. Both techniques have since been commercialized. His achievements have “lit the way” for research in optical metrology. The bond between Osten and Light Publishing Group can be traced back to 2012, when he first submitted a paper to *Light: Science & Applications* (LSA) as an author and published a paper^1^. Right after that, he was invited to join the Editorial Board for his enthusiasm. In 2019, he started the challenge of founding the new journal *Light: Advanced Manufacturing* (LAM) as the co-Editor-in-Chief. In this interview, Osten shared his insights on Optical Metrology, Holography, and the Founding of LAM with us. Follow the Q&As below and let us get closer to the “Holoknight”.

**Figure:**
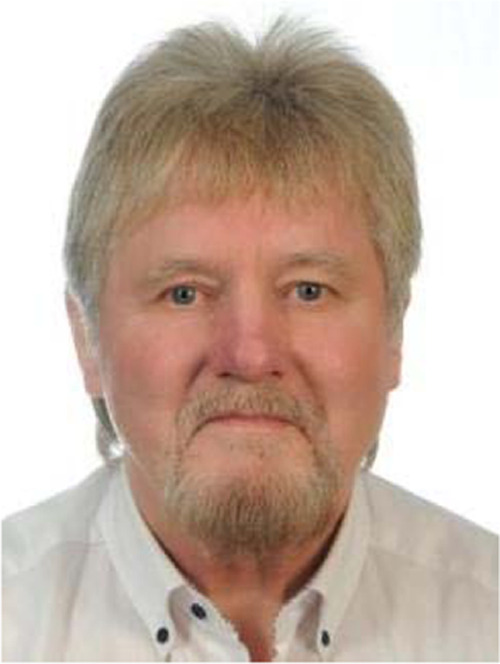

**Short Bio:** Dr., Prof. Wolfgang Osten received the MSc/Diploma in Physics from the Friedrich-Schiller-University Jena in 1979. From 1979 to 1991 he was working at the Academy of Sciences in Berlin in several institutes making investigations in coherent metrology, digital image processing, and machine vision. In 1983, he received a Ph.D. degree from the Martin-Luther-University Halle-Wittenberg for his thesis in the field of holographic interferometry. In 1991, he joined the Bremen Institute for Applied Beam Technology (BIAS) to establish and direct the department Optical 3D-Metrology till 2002. From September 2002 till October 2018 he has been a Full Professor at the University of Stuttgart and Director of the Institute for Applied Optics. From 2006 to 2010 he was the Vice-Rector for research and technology transfer at Stuttgart University. His research work is focused on new concepts for industrial inspection and metrology by combining modern principles of optical metrology, sensor technology, and digital image processing. Special attention is directed to the development of resolution-enhanced technologies for the investigation of micro and nanostructures. Wolfgang Osten is fellow of OSA, SPIE, EOS, and SEM, and senior member of IEEE. He is an Honorary Professor of Shenzhen University, China, an Honorary Doctor of the University of Technology of Ilmenau, Germany, the 2011 recipient of the Dennis Gabor Award of the International Society for Optics and Photonics SPIE, the 2018 recipient of the Rudolf Kingslake Medal of the SPIE, the 2019 recipient of the Chandra Vikram Award of the SPIE, and the 2019 recipient of the Emmeth Leith Medal of the International Society OPTICA.

**Q1. To my knowledge, you have been working on industrial inspection and metrology since the late 1970s, probably for your whole academic career. Would you like to tell us what made you interested in the study of this field?**

A1: That’s right. I studied physics at the Friedrich-Schiller University in Jena and the important content of our study was to learn to solve practical problems. In this context, all students had to complete an industrial internship and here I learned the advantage of joint work with industry partners to help them to improve the quality of their products. I could continue this kind of direct cooperation with the industry after leaving the university and working in the Academy of Sciences of the former German Democratic Republic (GDR) in Berlin. Besides the dedication to ambitious academic research, one important task of the Academy institutes was the scientific support of the industry. In this respect, the Academy was the large-scale research facility for the East-German industry. My diploma thesis and my later research were focused on optical metrology and inspection. Interferometry and image processing are powerful tools to understand the function of products and processes and find out how to improve them. The important need for quality control one can find in all areas of industrial production. This need, the big diversity of problems to be solved, and the close connection with the associated physical problems have always inspired my fascination for measurement technologies.

**Q2. Mendeleev said: “Science begins where one begins to measure,” and “exact science is unthinkable without measure.” Could you share with us your insights on the great importance of the accuracy of measurements?**

A2: There are so many wonderful sayings about metrology. Only to cite one more: Galileo Galilei said “It is necessary to measure everything that can be measured, and to try making measurable what isn´t yet.” Indeed, the accuracy of measurement is an important feature of any measurement. That’s why it gives us an idea of our measurement and tells us the truth about the object under test. However, judging the accuracy is not even simple. Usually, we look for the uncertainty or precision of a measurement result. For the calculation of that feature, there are well-established procedures that are controlled by the national institutes of standards such as the National Institute of Metrology (NIM) in China, the National Institute of Standards and Technology (NIST) in the US, and the Physikalisch-Technische Bundesanstalt (PTB) in Germany, There is an internationally accepted framework for the expression of uncertainty in measurement—the GUM guidelines^2^.

**Q3. Optical Metrology has been evolving during the past century, what is the latest challenge for metrology equipment? Looking into the next decade or even the next century, what are the major challenges and opportunities for Optical Metrology?**

A3: Indeed, metrology has a long tradition. Remember my citation of Galileo Galilei above. However, the increasing complexity of products and the continued miniaturization of the critical structures, which are responsible for their function—as we can see especially in nano- and semiconductor technology—create new challenges that need to be mastered in order to assure the quality of future products. One of the biggest challenges is the enhancement of the resolution of optical measurements. Optical principles have unfortunately obvious physical limitations here and it needs a lot of tricky measures to overcome them. Especially, the implementation of effective procedures that deliver a high-resolution and high-precision measurement on extended functional surfaces is a challenge that gives us enough work for the coming years. Another interesting topic is the inspection of organic material where scattering, absorption, translucence, … occurs. This kind of measurement object can be found for instance in medical technology where procedures are searched for the intra-operative identification of tissue properties, which can be used for the reliable distinction between malign and benign tissue parts.

**Q4. From 2006 to 2010 you were the Vice-Rector for research and technology transfer at Stuttgart University. We believe that you have a close connection with the industries. From your point of view, what is the biggest difference between fundamental research and the industrial applications of the same technology? How to effectively build a bridge from scientific research to the industrial application?**

A4: The biggest difference is that the industry is not only asking for printed and published papers. The industry is looking for practical and verifiable results that can be applied for the direct improvement of the fabrication processes and even more the fabricated products. Of course, a deep physical and technical understanding of the process, the function of the entire product, and/or its deciding parts is the fundamental basis of all practical implementations and verifications. However, for partners in the industry, the proof of the truth is done, when it is shown that it works. I like the saying made by W.E. Deming: “In god we trust, all others have to bring data!” The mentioned bridge is not even simple to build. But here in Germany, we made very good experiences with the so-called collaborative research that is organized by the German Federal Ministry of Education and Research. This kind of project happens through dense cooperation between academic researchers and experts from the industry. This is an already long-term successful measure in Germany and has anticipated what people in the US “invented” a couple of years ago and called “Open Innovation”.

**Figure:**
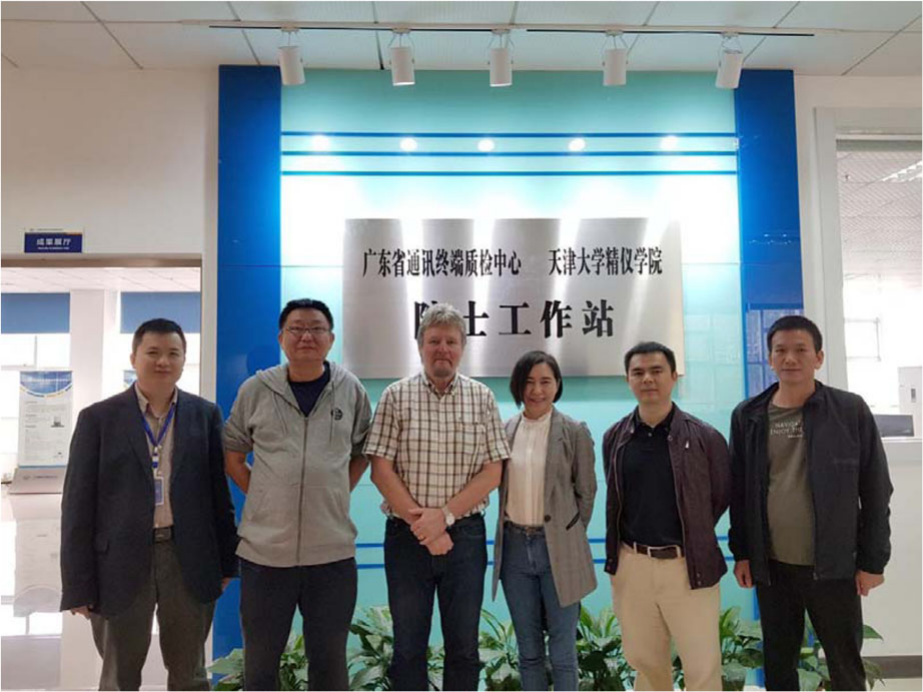
On occasion of a visit of the Shenzhen University in 2017 with Prof. Yuhong Bai, Prof. Guohai Situ, and Prof. Xiang Peng

**Figure:**
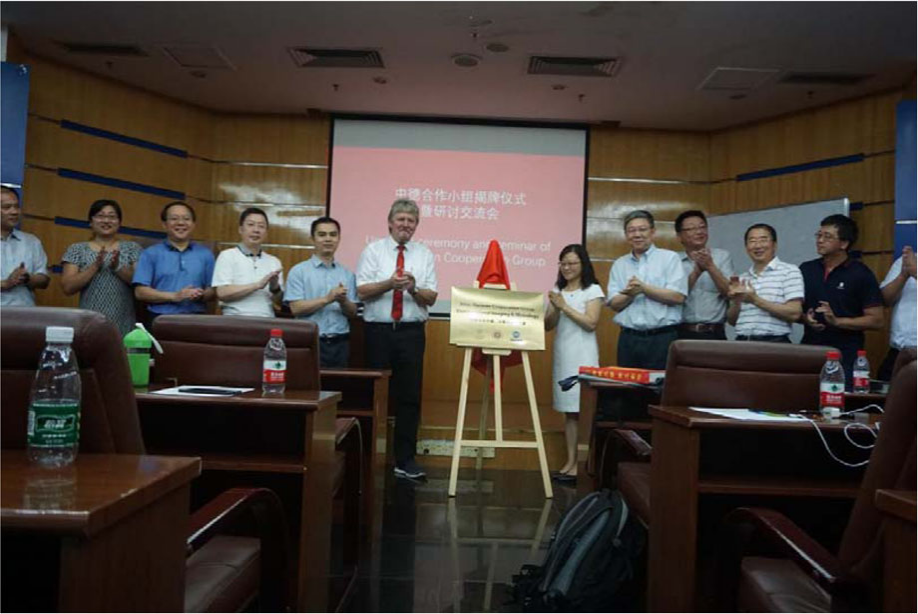
Opening of the Joint Shenzhen Univesity and Stuttgart University Research Center for Computational Imaging supported by the Chinese Science Foundation and the German Research Association in 2018

**Q5. According to the European Union Industry 5.0, the new concept of Industry 5.0 provides a vision of industry that aims beyond efficiency and productivity as the sole goals and reinforces the role and the contribution of industry to society. How will it change or affect our lives?**

A5: Industry 4.0 was a measure to support the wide implementation of digital technologies in modern industrial production. Therefore, the focus of that program was clearly directed to the improvement of the competitiveness and efficiency of the European industry. The new initiative, Industry 5.0, has a completely different approach. Industry 5.0 provides a vision of an industry that aims beyond efficiency and productivity as the sole goals and reinforces the role and the contribution of industry to society. It places the well-being of the worker at the center of the production process and uses new technologies to provide prosperity beyond jobs and growth while respecting the production limits of the planet. This is a strict paradigm change. Not the product is at the center of all activities but the producer and customer, respectively. I think that this change is a consequence of the altered boundary conditions that confront the entire mankind with new challenges: climate change, environmental pollution, lack of resources, aging society, aging infrastructure, and unfairness in the worldwide distribution of welfare and wealth, … I really hope that this are not only big words but that the program contributes to a real paradigm change and acts as a kind of game changer in our understanding of the real qualities of life such as an intact environment, good and stable social structures, a safe and healthy existence for everybody, and a fair distribution of the resources of our planet.

**Q6. You have won many international awards such as the Dennis Gabor Award, the Rudolf Kingslake Medal, the Chandra Vikram Award, and the Emmeth Leith Medal. What do these awards mean to you?**

A6: For me, they do not mean more and no less than that my esteemed colleagues think quite positively about my results achieved.

**Q7. You have trained many PH.D. students in your career, mentoring their work, and guiding them when they lose confidence and doubt themselves at the beginning of their research. Do you have any advice for students and young researchers?**

A7: That’s a very complex question. And there are no general recipes to offer. However, the most important for me in all discussions was to try to find a common answer to the simple question “What is the objective of your study, what do you wish to achieve finally after all the effort that you have done?” That means for me the importance of robust motivation. When this is clear, the way to achieve that goal is often well defined.

**Figure:**
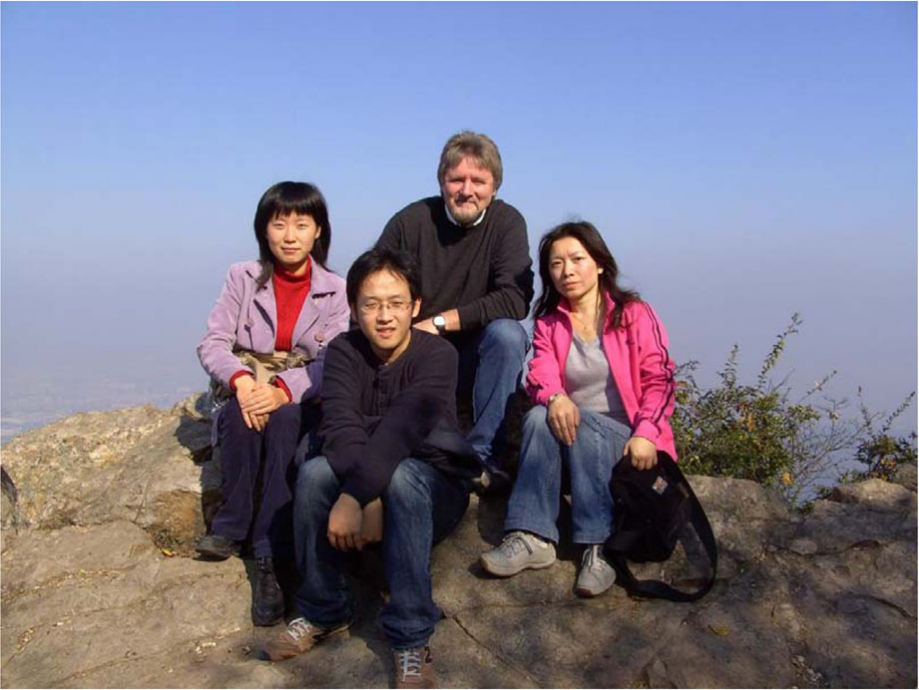
With Prof. Cherry Che and Dr. Yun Ma of the Nanjing University on occasion of a Visit of the Nanjing University in 2008

**Q8. You were the co-chair of Light Conference 2019 in Changchun, China. What do you expect from activities such as the annual Light Conference? What should we bring to the audience in Light Conference Week 2022 (celebrating the 10th Anniversary of Light)?**

A8: Oh, 2022 is almost over. Maybe we think about 2023? For me, the deeper sense of international conferences is to strengthen the communication and understanding between scientists coming not only from different countries but even more with a different cultural backgrounds. We call it diversity that makes our social connections richer. The open exchange of new findings and ideas is the best way to preserve our common planet as a peaceful spaceship for all its passengers. My experience with the Light Conferences is very positive in that sense. The atmosphere created by the organizers is very open, friendly, and stimulating. The contact with young Chinese scientists is very pleasant and shows the continuously growing quality standard of their research. However, the next conference—4 years after the last one in 2019—will take place in a world that has changed considerably. The Covid pandemia and the grown international tensions will make it not even easier to organize a conference with the spirit of the last Light Conferences. Thus it is our joint goal to find back to normality and build a bridge that gives all participants the feeling that they are welcome and invited to make our research a common asset that contributes to health, peace, and prosperity for all countries. The Light journals can play an important role in that process as a kind of diplomat, far from economic interests and dedicated to open and peaceful communication among scientists.

**Figure:**
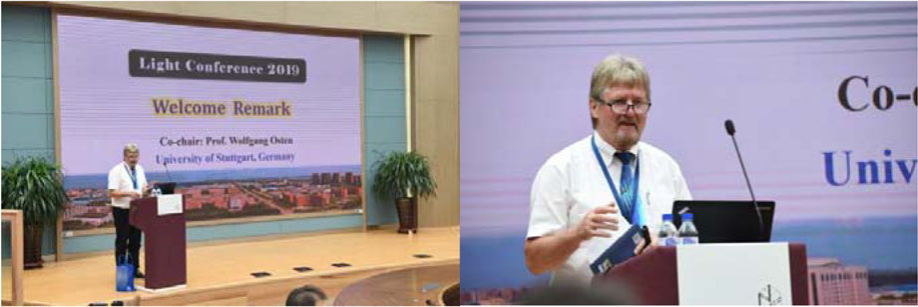
Prof. Wolfgang Osten gave a welcome remark at Light Conference 2019 as the Co-chair

**Q9. In 2019, you accepted Prof. Yuhong Bai’s invitation as the founding Editor-in-Chief of Light’s sister journal LAM and built this new journal from scratch. What factors and opportunities made you willing to accept this challenge? What are your vision and expectations for the new journal?**

A9: First of all it was my very positive experience with the cooperation with the CIOMP people for the *Light: Science and Applications* journal that convinced me that the joint LAM-project will be successful. Especially, I wish to mention here the very effective and friendly cooperation with Prof. Yuhong Bai and Chenzi Guo. Furthermore, the concept of LAM corresponds with my above-mentioned opinion: making science work for the benefit of people by fruitful cooperation with the industry. We had a long discussion about the unique position of LAM in comparison to already existing journals with a similar focus. Here we found out that LAM could fill a space by bridging the gap that often exists in moving technologies to market. A journal with a wider scope in manufacturing technologies but with a clear focus on photonic technologies was still missing. All these steps were done in a very good atmosphere and the cooperation with the LAM team was gratifying and effective from the beginning. My vision is that together we create a new scientific journal of high quality that will take a visible place among a large number of existing journals. For the measurement of the success of a journal, there are clear indications such as the impact factor. But for me, it will be a first positive indication when acknowledged colleagues choose the LAM journal for the publication of their latest findings.

**Figure:**
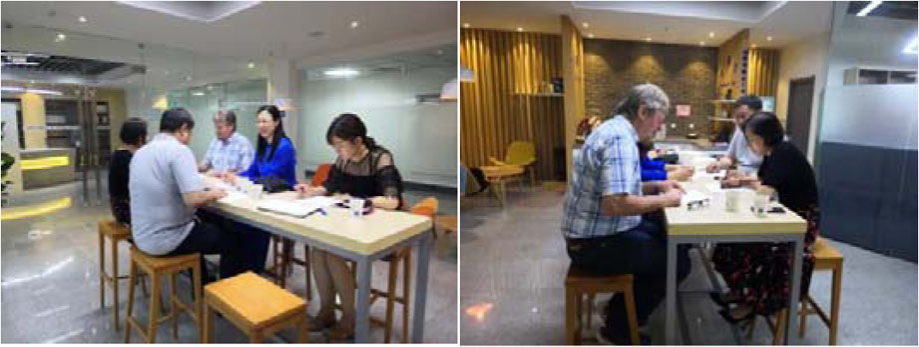
Prof. Wolfgang Osten was discussing the establishment of LAM with the Publisher and the Organizer

**Figure:**
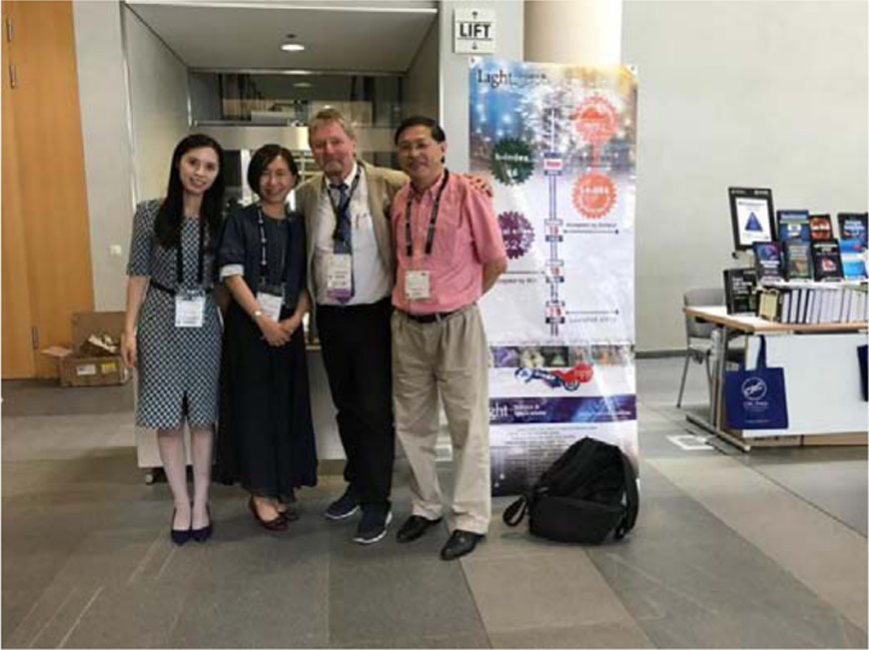
Dr. Chenzi Guo, Prof. Yuhong Bai, Wolfgang Osten and Min Gu at Laser World of Photonics 2017

**Q10. What has been your biggest challenge so far in starting LAM? What characteristics do you think a good journal should have? What is your strategy for building LAM? What do you think is the most important thing about running a journal?**

A10: The biggest challenge was to create a good concept and to find an editorial board where the members are not only present by their name but work actively for the success of the journal. This could be completed together very soon. A good journal stimulates authors to accept it as their most preferred journal for the documentation of the success of their scientific activities. A good journal has not had to make too many advertisements but impresses by its quality and especially by the support of the editorial office and a fast as possible publication. A good journal always convinces the authors that their publication is not only for the benefit of the journal but mainly for their own benefit. My strategy: to conquer LAM a permanent and recognized place in the concert of all related journals. Most important: to give the authors always the impression that this journal works for them and also important to have enough energy for the long run. That’s especially because the competition among all existing journals is very strong and every day new journals are created.

**Q11. The marriage of the holographic and laser principle opened the gate for a large variety of new technologies and applications in optical imaging, information processing, and metrology. In 2021, you initiated the “Featured Issue on Celebrating Holography after 60 years of successful application” of** ***Light: Advanced Manufacturing*. Also, you have contributed one Review for this collection to summarize 55 Years of Holographic Non-Destructive Testing and Experimental Stress Analysis**^3^. **What is the special significance of this technology? Where do you see it will go in the future?**

A11: The special significance of holographic technologies for Holographic Nondestructive Testing (HNDT) is hopefully clearly explained in the preface of the special issue and the mentioned article. Holography opened the door for the investigation of a variety of objects having technical surfaces. This opportunity was very limited with classical interferometry which has its strength for the investigation of the shape of objects having smooth surfaces such as lenses and mirrors. HNDT widened the application range for interferometric techniques beyond that limited application. It allows to the investigation of real industrial products with respect to their response to any artificial or operational load. Relevant examples are the monitoring of vibrations of a big bridge while a train is crossing, the inspection of the vibration behavior of automotive engines, and the sound generation across the car body where the engine is working. Only this way, we can see what we hear for example. About the future. Another saying tells us that “*The future is open and confusing*”. However, I am optimistic with respect to the relevance of holographic technologies for the creation of new products and the solution of challenging practical problems. Holography and related technologies such as Speckle techniques find more and more acceptance in various fields. To name just a few: the implementation of real 3D screens beyond the conventional stereoscopic principle, the minimum invasive investigation of cultural heritage and artwork as a valuable tool for conservators, the inspection of large-scale facilities with respect to material weakness, corrosion, and internal faults. However, as Charles Vest already mentioned 40 years ago in his inspiring study for the National Bureau of Standards of the US^4^, the technology is still confronted with a bunch of challenges before to be accepted as a reliable measuring and inspection tool. To them belongs especially the in-line performance, meaning the ability to work not under special laboratory conditions but nearby the manufacturing process. Further everlasting requirements from the industry are increased robustness against environmental disturbances, improved user-friendliness and resolution, and the performance for studying volume scattering and translucent materials. There is a nice recent publication where a lot of future activities are listed^5^. The fulfillment of all these wishes provides enough material for future research.

**Figure:**
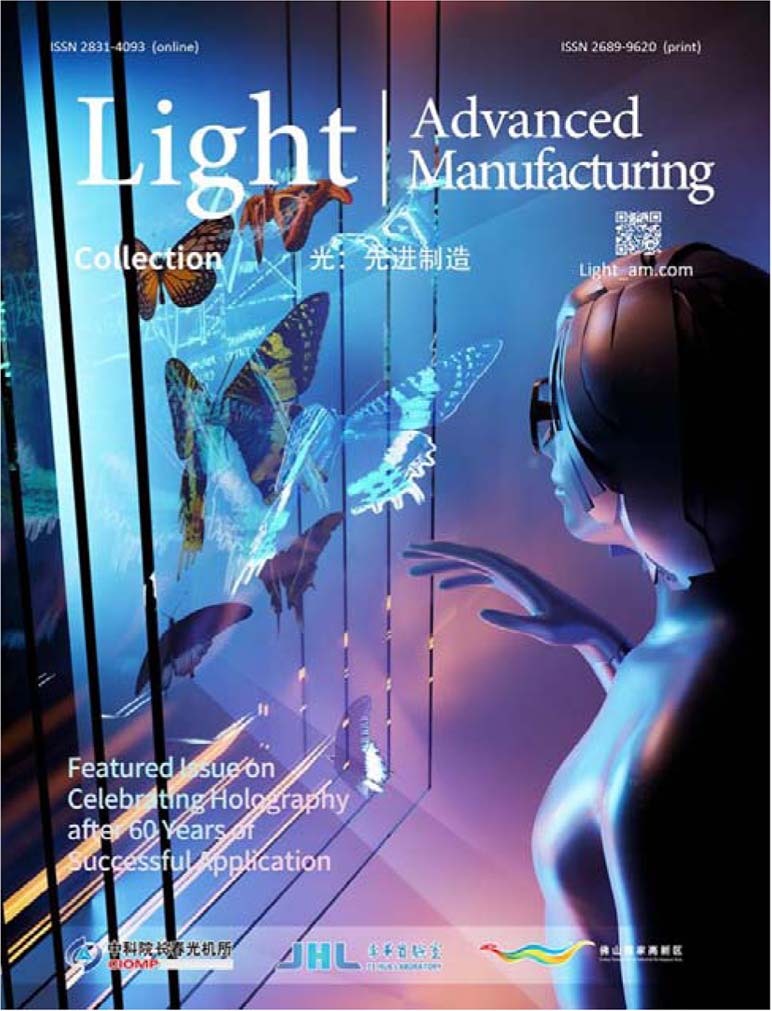
Cover of Featured Issue on Celebrating Holography after 60 Years of Successful Application
